# Obedience induces agentic shifts by increasing the perceived time between own action and results

**DOI:** 10.1038/s41598-024-66499-8

**Published:** 2024-07-22

**Authors:** Nil Akyüz, Hans Marien, F. Marijn Stok, Josi M. A. Driessen, John de Wit, Henk Aarts

**Affiliations:** 1 Department of Psychology, Utrecht University, PO BOX 80140, 3508 TC Utrecht, The Netherlands; 2 Department of Interdisciplinary Social Science, Utrecht University, Utrecht, The Netherlands

**Keywords:** Psychology, Human behaviour

## Abstract

The concept of 'agentic shift,' introduced by Stanley Milgram, suggests that obedience reduces the sense of agency. In a recent study simulating the seminal work of Milgram, Caspar et al., 2016 examined this idea in a financial harm context. They demonstrated that, compared to situations of voluntary decision-making, coercion increases the perceived time between action and outcomes—suggested as a marker of diminished agency. Importantly, in this study, participants were agent and victim (relying on a reciprocal relationship) and first experienced free choices, followed by forced choices. This diverts from Milgram’s original study, where participants were no victims but only agents who were forced to harm. The current study replicates and extends findings from the 2016 study by Caspar et al. in an online meeting setting, where participants served only as agents—similar to the original Milgram studies—while controlling the order of free and forced choice blocks. Substantiating earlier findings, forced choices reduced temporal binding (increased time interval estimations) compared to free choices independent of the order. We briefly discuss the importance of replications of coercion effects on the sense of agency, particularly in online decision-making settings.

## Introduction

When assessing the moral aspects of an action, it is crucial to consider the extent of agency an individual feels, particularly in the presence of external pressure. A pivotal study addressing moral responsibility, conducted by Stanley Milgram, offers a compelling foundation for this discourse by examining the dynamics of obedience. In Milgram’s study, participants received orders from an authority figure to administer electric shocks to a confederate^1^. A majority of the participants delivered shocks up to the maximum level despite being aware that they were causing harm to others, obeying the experimenter's orders. Milgram introduced the term "agentic shift" to describe this phenomenon: The agentic state was deferred to the authority issuing the orders, while participants claimed they were "only following orders"^2^.

Although the agentic shift offers an explanation for harmful actions inflicted upon a defenseless victim, participants who acted as offenders might report a reduced sense of agency as a tactic to evade blame or repercussions, thereby achieving additional benefits such as avoiding punishment or lessening their responsibility^3^. To prevent potential bias of such strategic self-reporting, Caspar et al.^4^ proposed the use of implicit markers as a more appropriate indicator of agency. One such implicit marker of agency is perceived temporal binding^5–9^. Temporal binding refers to the phenomenon where an action (e.g., pressing a key to harm someone) and its subsequent outcome (receiving audio input representing the harm) are perceived to occur closer in time when the action is voluntary and freely chosen compared to involuntary action, i.e., being forced by an external cause. Accordingly, coercion could decrease temporal binding.

In a study adapting the temporal binding measure to the Milgram setup, Caspar et al.^4^ invited two participants at the same time to the test setting. They were tested in pairs and took turns being “agents” and “victims”, ensuring reciprocity. In one group of pairs, the agent could freely choose on each trial to earn money by taking money from the “victim”, while the roles were reversed in a second round. This free-choice condition was compared to a subsequent coercive condition, in which the experimenter stood next to the agent, ordering before each trial whether to take money or not. This procedure was repeated with another group of participants in the context of gaining money by administering an electric shock (physical harm) to the “victim” (see Ref.^10^ for a further account of this novel experimental approach to study disobedience to authority). The results showed a decrease in temporal binding in the coercive conditions, independent of the type of harm. While the findings suggest that coercion indeed increases the perceived time between own action and results, there are a few aspects that divert from the Milgram setup, preventing a firm conclusion about obedience effects on temporal binding. In the present study, we aimed to address these issues in a replication of the financial harm experiment.

Primarily, we reevaluated the reciprocity aspect in Caspar et al.'s^4^ experimental design, which involved role reversals between the agent and victim roles of participants. In this reciprocal setting, participants who were first victims and then agents may use a different motivation or strategy to punish the previous agent, such as retaliation or plain imitation. Caspar et al.^4^ found that participants inflicted a similarly high number of harms when they had previously experienced harm themselves. Such copying of behavior raises questions about the true nature of free choice^11^. Our study, therefore, employed a counterfeit participant in the victim role, ensuring that participants always acted as the agent. This crucial adjustment also more closely aligns with the unidirectional authority-subject dynamic observed in Milgram's original study^1^.

Furthermore, we introduced two other features concerning the test setup. First, in the earlier study on coercion and temporal binding^4^, free-choice trials always came before the coercive trials, neglecting to consider the potential effects this sequence could engender (but see Refs.^12,13^). For example, starting with free choice renders the forced choice condition less pleasant, as losing personal autonomy is commonly experienced as annoying^14^. As a result, the effects of free (vs. forced) choice on the sense of agency might be strengthened. However, different reasoning might hold for the reverse order, in the sense that starting with coercion might undermine the understanding to choose freely, thus weakening the effect of free choice on temporal binding. It is important to note that in the original Milgram experiment, participants were always coerced and not provided with a free-choice option, thus diverting from the order implemented by Caspar et al.^4^. To empirically investigate the potential effect of the order, we manipulated the sequence in which participants were coerced or free to harm another person.

In addition, we aimed to address the physical presence of the experimenter during the experiment, as was the case in Caspar et al.^4^. This presence could lead to experimenter effects and biases that influence behavior of the experimenter and participant (e.g., the experimenter may act differently and influence the experiences or behavior of participants in line with the hypothesis) beyond the intended objective manipulations (e.g., Ref.^15^). Consequently, in the present study, participants received instructions via an audio recording following a strict protocol, while the experimenter was entirely out of sight and not involved in any part of the experiment. Acknowledging the significance of a double-blind design in recording any psychological variable^16^, this approach was intended to minimize any potential biases of experimenter effects on the result.

In sum, our study presents three significant modifications to the Caspar et al.^4^ experimental design: the elimination of reciprocity, the counterbalancing of free choice and forced choice order, and the physical absence of the experimenter. It is important to note that the current experiment was conducted amidst the severe restrictions and measures on laboratory use and participant testing due to the COVID-19 pandemic in 2021 and 2022 in the Netherlands. Due to the restrictions imposed by the COVID-19 pandemic, the study was carried to an online meeting platform, this shift provided us the opportunity to investigate the effect of choice restrictions in online environments. In doing so, our test might offer an important application to online settings that have become common practice in social interaction.

## Results

### Descriptive behavioral statistics

In total, financial harm was given in 638 out of 2516 free trials recorded (15.2/60 trials, CI_95_ = [9.82, 20.56], min = 0, max = 60). Among 42 participants, one participant always chose to press the button that caused financial harm, and 11 participants never chose to cause financial harm. The remaining 30 participants caused financial harm in some trials.

The results of a chi-squared test indicated a significant association between block order and the decision to take or not take money, χ^2^ (1, N = 2516) = 157.51, *p* < 0.001. Participants who started with the free block were more likely to choose to take money (in 36% of the free trials) than the participants who started with the forced block (in 14% of the free trials).

### Perceived autonomy and emotions

**Figure 1:**
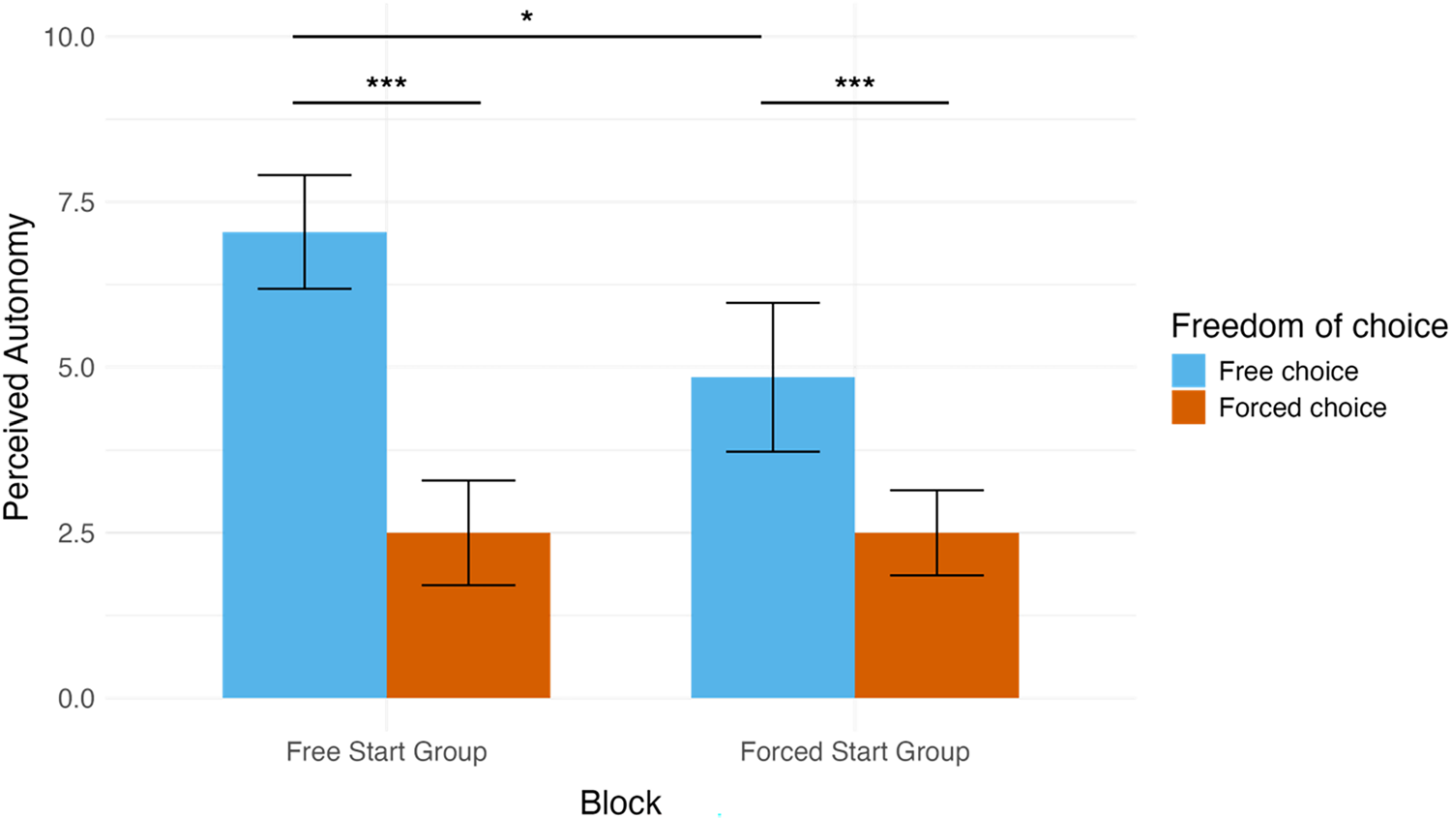
Perceived autonomy scores by freedom of choice and block order. The mean and standard error values of the perceived autonomy scores for free and forced conditions are displayed by block order. The left side illustrates the pattern when free choice trials were presented first, and the right side shows data when forced choice trials were presented first. *p < 0.05, **p < 0.01, ***p < 0.001.

A repeated-measures ANOVA was conducted to examine the differences in perceived autonomy between the free and forced conditions and included block order (starting with free vs. forced block) as the between-subject factor (Fig. 1). The results revealed a significant main effect of freedom of choice, F(1, 40) = 67.77, *p* < 0.001, η_p_^2^ = 0.63. Participants felt more restricted in their autonomy in the forced (M = 2.50, SD = 1.69) than in the free condition (M = 6.00, SD = 2.54), t(41) = 7.83, *p* < 0.001, Cohen's d = 1.62. There was also a significant main effect of block order (F(1, 40) = 5.52, *p* = 0.024, η_p_^2^ = 0.12). The interaction between freedom of choice and block order was significant (F(1, 40) = 6.87,* p* = 0.012, η_p_^2^ = 0.15). As the post-hoc-pairwise comparisons with Bonferroni correction indicated that participants experienced less autonomy in the free choice trials in the group that started with forced trials (M = 4.85, SD = 2.56) compared to the participants who started with the free trials (M = 7.04, SD = 2.06), t(40) = 2.35, *p* < 0.024, Cohen's d = 0.94.

Additional separate ANOVAs were conducted to assess the effects of freedom of choice (within-subjects factor) and block order (between-subjects factor) on the emotional experiences (enjoyment, struggle, and unpleasantness) of the participants. These experiences were reported by participants at the end of the task for free and forced conditions separately. None of these analyses revealed significant main or interaction effects (detailed results for these ANOVAs are provided in the supplementary material).

### Time interval estimation (TIE)

The analysis of the linear mixed model fit (see "Method" section below) revealed a significant effect of freedom of choice on time interval estimations (β = − 11.56, SE = 3.78, df = 4994.83, t = -3.06, *p* = 0.002). The time interval estimation was larger in the forced (M = 369.75 ms, SD = 121.40) than in the free-choice condition (M = 348.45 ms, SD = 129.77). There was no significant main effect of outcome (β = − 3.76, SE = 3.94, df = 5012.98, t = -0.95, *p* = 0.34) or block order (β = 1.51, SE = 18.44, df = 40.97, t = 0.08, *p* = 0.94).

**Figure 2:**
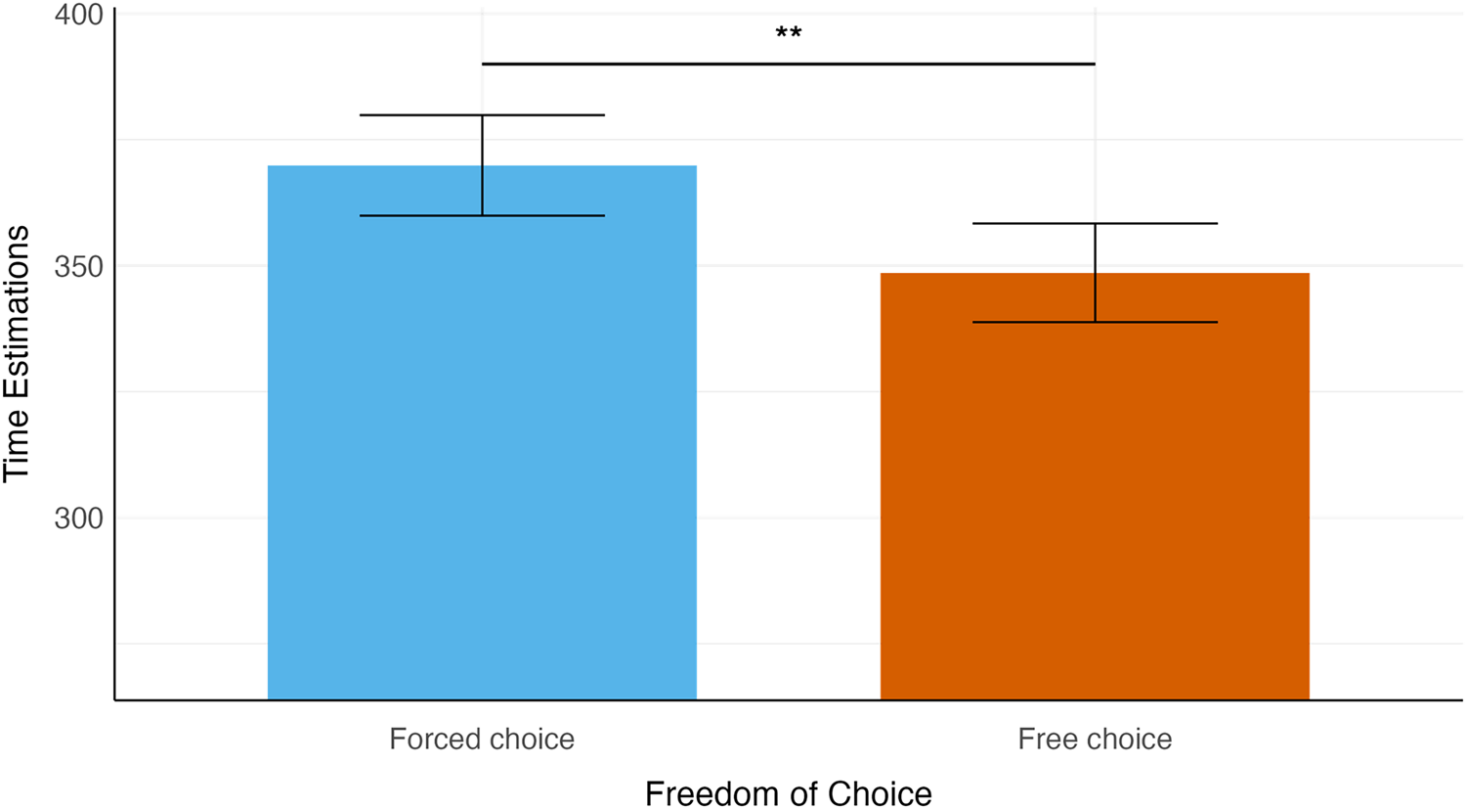
Time interval estimations by freedom of choice. The means and standard error bars of the means for the time interval estimations in the free and forced conditions. *p < 0.05, **p < 0.01, ***p < 0.001.

When looking at the interaction effects, neither the interaction between freedom of choice and outcome (β = 0.83, SE = 3.94, df = 5012.84, t = 0.21,* p* = 0.83), nor between freedom of choice and block order (β = − 0.68, SE = 3.78, df = 4994.83, t = − 0.18, *p* = 0.86), or between outcome and block order (β = -5.49, SE = 3.94, df = 5012.98, t = − 1.40, *p* = 0.16) were significant. Finally, the three-way interaction between freedom of choice, outcome, and block order was also not significant (β = − 1.18, SE = 3.94, df = 5012.84, t = − 0.30, *p* = 0.76). Figure 2 presents the means and standard error bars for each cell in the experimental design.

Additionally, to provide a proxy for a between-subjects experimental design, we conducted a supplementary analysis on the first block responses. See the supplementary material (Supplementary Analysis on TIE for First Block Responses) for the detailed analysis.

## Discussion

Our primary objective was to test the influence of coercion on sense of agency, revisiting the original work of Milgram. We adopted the temporal binding measure of agency, in line with the coercion method used by Caspar and colleagues^4^. Importantly, the method does not directly address or test the Milgram effects in itself, but it offers a nice, experimentally controlled set up for simulating the Milgram experiments. However, diverging from their method, we excluded the reciprocity dimension from the time interval estimation protocol. Our method always incorporated the same victim, ensuring that participants solely functioned as agents, inflicting harm without the prospect of receiving it. This design concurs with the unilateral authority-subject dynamics typical for Milgram’s seminal 1963 study. Echoing Milgram’s foundational work, our data revealed longer time interval estimations between own actions and their results in the forced condition compared to the free choice condition. This divergence in temporal perception speaks to the phenomenon of the agentic shift.

Notably, our findings resonate with the first study of Caspar et al.^4^ and subsequent studies using the reciprocity context^12,17^. Even after eliminating the reciprocity element, which might introduce potential carry-over or automatization effects, we found a coercion effect on sense of agency. In addition, and similar to Caspar et al.^4^, our results demonstrated that the type of action outcomes (harm or no harm) did not significantly alter the perceived time intervals, underscoring the primacy of decision-making autonomy and freedom of choice over the action outcome in shaping agency^18,19^. Furthermore, our replication reinforces the perspective of Haggard and Chambon^20^, which posits that agency emerges as a metacognitive experience that is deeply rooted in the act of decision-making. Their perspective asserts that agency goes beyond mere sensorimotor outcome prediction suggested by the comparator model^21,22^. Our study, showcasing the shift in agency based on freedom of choice, substantiates this broader perspective, emphasizing the pivotal role that choice holds in shaping the perception of our own behavior^23–25^.

Furthermore, our study aimed to examine two other aspects that are important to further scrutinize in the effects of obedience on temporal binding. First, we were able to rule out order (free vs force choice) effects; it did not matter whether free choice or forced choice was measured as first. This aligns with more other recent work on obedience and temporal binding^12,13^. Interestingly, the effect of order was important for the subjective perceived autonomy of the participants. While coercion weakened perceived autonomy, this effect was more pronounced when free choice was followed by forced choice. These findings suggest that losing autonomy (when moving from free to forced choice) has a more notable effect on subjective experiences of control than gaining autonomy (when moving from forced to free choice). This residual effect demonstrates how context can affect our explicit feelings of autonomy under changing circumstances. Such an observation resonates with the basic need principle proposed by self-determination theory^26^. The perturbation in one's perception of a fundamental need, such as personal autonomy, can recalibrate subsequent perceptions and motivations of behavior related to that need^27^.

Secondly, in our study we took away the role of the experimenter. In the Caspar et al.^4^ study, the experimenter stood directly beside participants and issued commands. Previous research suggests that an experimenter’s presence can influence the behavior of individuals, particularly in contexts in which the experimenter is part of the study that involves harm, as highlighted by Caspar, Gishoma, and Magalhaes de Saldanha da Gama^28^. This influence could be due to experimenter effects, which may amplify the effect of freedom of choice on the temporal binding (TB) measure. In line with the present setup, later studies^13,28–30^ addressed this issue by employing audio recordings to reduce direct interactions with the experimenter and to rule out experimenter effects. However, whereas these studies divert from the initial Caspar et al.,^4^ setup in that they examined a diversity of other aspects of obedience and measured different aspects of agency experiences, the fact that we replicate the choice effect in the absence of an experimenter further indicates the direct impact of freedom on decision making and awareness of action*.*

There are a few limitations in this study that we wish to address. One important limitation was the absence of a manipulation check regarding participants' belief in the cover story of financial game. In the absence of this control, it is difficult to eliminate the effect of participants' skepticism with the cover story which might affect their responses in various ways. For example, participants who doubted the cover story could have been subject to desirability bias, which would have led them to avoid harming the counterfeit participant^31^. To further understand its influence on study findings, manipulation checks should be incorporated into future research to examine participants' trust in the cover story. Additionally, our research was conducted during the global COVID-19 pandemic, forcing us to resort to research methods that excluded physical contact with participants. Therefore, in contrast to many temporal binding experiments^4,12,17,23^, our study was conducted in an online communication setting. The online setting was less controlled compared to a lab environment considering the environment in which the participants were partaking in the experiment. The uncontrolled environment may introduce variability in participants' responses, likely creating noise in the data and leading to an underestimation of the effect size.

While the online setting might introduce additional variability in the data, it also eliminated potential undesired effects that might stem from the experimenter’s presence. Online testing not only optimally accommodates the prevailing circumstances but also reflects the escalating significance of digital interfaces in modern research landscapes^32^. By stepping into the territory of online communication and experimentation with time interval estimations, we emphasize the salience of the availability of choice on digital platforms. As the digital realm expands, online autonomy is rapidly becoming a cornerstone for shaping perceptions of agency^33^. However, online systems that are crafted by developers or driven by algorithms can inadvertently or deliberately restrict user choices and thus may cause agentic shifts. By exploring online experimentation with time interval estimations, we underline the pressing need for future research to study how freedom of choice changes the way we perceive our own behavior in online environments and the virtual world.

In conclusion, our study replicates the findings of the seminal work from Caspar et al.^4^. Using the TIE measure as an implicit marker of agency, we demonstrated that the time passing between an action and its effect is perceived as longer when participants were forced (vs. free) to take money away from another person, thus causing harm to the person. We also observed that the effects of the TIE measure coincided with perceived autonomy scores. Participants reported lower perceived autonomy in forced trials compared to free trials. This underscores the importance of objective states of personal autonomy in shaping agentic experiences.

## Methods

### Ethics approval

The study was approved by the Human Ethics Review Board of the Faculty of Social and Behavioral Sciences, Utrecht University (application number 20-537). Specifics of the experimental setup, including the use of online meetings, were outlined in the application. This research was conducted in accordance with the principles of the Declaration of Helsinki. All methods were performed under the relevant guidelines and regulations. Informed consent was obtained from all participants.

### Participants

Forty-five participants (29 female) completed the experiment. They were recruited from SONA (Utrecht University's Social Sciences Research Participation System), the Prolific.sc recruitment website, and student social media groups by sharing an online flyer. The flyer contained a link to a Doodle survey for participants to indicate their preferred time slots. Based on their responses, we dispatched Microsoft Teams invitations for the online meetings. The inclusion criteria required participants to be native Dutch speakers and over 18 years old. All participants signed informed consent before the experiment. Random ID numbers were used to ensure the confidentiality of the data. The current sample size of n = 45 would allow us to detect an effect of choice on TIE of Cohen’s dz = 0.43 (with a power of 0.8 and an alpha level of 0.05). This is comparable to the sample size of n = 42 and effect size Cohen’s dz of 0.5 established in the financial harm experiment of Caspar et al.,^4^.

### Design and procedure

The study had a 2 (block order: free-first vs. forced-first) × 2 (freedom of choice: free vs. forced) × 2 (outcome: harm vs. no-harm) mixed factorial design, with block order as a between-subjects factor and freedom of choice and outcome as within-subject factors. The procedure closely followed that of Caspar et al. (Ref.^4^, Experiment 1), with the important notable difference that it was executed in an online meeting session. The experimenter explained the financial game to participants, positioning them in active roles where they had the opportunity to deduct money from a passive participant. All participants were granted a starting amount of 5 euros. Throughout the task, the active participant could decide to make monetary deductions in small increments. Importantly, once these preliminaries were discussed, the experimenter explicitly advised against the use of Bluetooth accessories and then exited the online meeting. This ensured that the experimenter was absent while participants took part in the experiment, fostering an environment such as the autonomous setting of online tasks. Participants were then directed to the Gorilla.sc platform to start the experiment.

Participants were randomly divided into two groups. One group began with the free-choice trials, while the other started with the forced-choice trials. After completing the initial 60 trials, each group transitioned to the alternate condition, ensuring that all participants experienced both types of trials. After the time interval estimation task, participants were asked to report their perceived autonomy and emotions in different conditions. Finally, they were asked to provide demographic data. Upon conclusion of the task, they were redirected back to the Teams meeting with the experimenter, debriefed about the confederate, and provided with their compensation. Participants were not inquired about their belief in the cover story, which is a limitation of our procedure. The entire procedure lasted approximately 30 min. Participants' final compensation ranged between 5 and 9.50 euros, contingent upon decisions made during the task. All the information provided and the questionnaires used, as well as the overall experiment, were in Dutch.

### Perceived autonomy and emotions

To check the freedom of choice manipulation, participants were asked to report their perceived autonomy restriction in both free and forced choice conditions. The perceived autonomy question was adapted from an earlier study^34^, and further tested and validated in a recent study aimed at optimizing the TIE task in a natural setting^35^. These questions assessed experiences of autonomy restriction, enjoyment, struggle, and unpleasantness during each condition on a Likert scale from 1 (not at all) to 9 (very much). For perceived autonomy, participants were asked to rate their feelings of autonomy restriction in both free and forced choice conditions. Afterwards, we reverse-coded responses; scores represent greater perceived autonomy. We also included two other questions referring to the naturalness of the TEAMS setting, but these are not relevant to the present test. We were primarily interested in the perceived autonomy question but also looked at the enjoyment, struggle, and unpleasantness changes as a sign of effects on emotional experiences and report our finding related to these emotional experiences in the Appendix.

### Time interval estimation (TIE)

The TIE task assessed participants' perception of the time interval between a keypress action and its subsequent auditory outcome. Participants had the option to press either the "X" or "M" key. Pressing one key resulted in a monetary deduction from a passive participant (cf., a 'victim participant’), whereas the other maintained the current balance. The mapping of the keys to actions varied randomly across participants. The outcome of their action was signified by a 500 Hz tone and an updated balance display.

In the free-choice trials, participants could select their desired key. Conversely, in the forced-choice trials, they were needed to follow instructions and press the designated key. Before the onset of each trial, a screen displaying balance information was shown for 2000 ms. This was followed by a cue screen indicating the type of trial. 'Kies' (Dutch for 'choose') denoted a free trial, while 'Gedwongen' (Dutch for 'forced') was accompanied by an audio message indicating the nature of the forced trial. This audio message was a male voice recording, either instructing "Pak het geld!" (Take the money!) or “Pak niet het geld!” (Do not take the money!). The free trial cue remained on the screen for 750 ms, while the forced trial cue was displayed for 1000 ms, as it also included the audio instruction.

**Figure 3:**
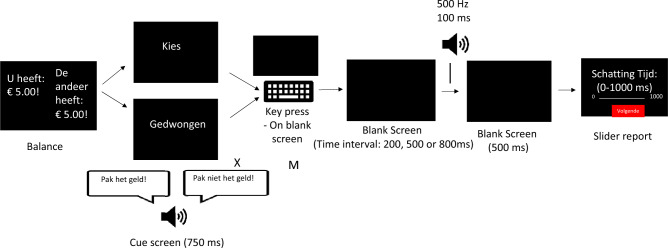
Flow Chart of Experimental Procedure. Participants start by seeing the balance screen ("U heeft"—You have, "De ander heeft"—The other has). Then they perform either a free ("Kies") or forced ("Gedwongen") choice. In free choice trials, they select to take ("X") or not take ("M") money. In forced choice trials, they hear either "Pak het geld!" (Take the money!) or "Pak niet het geld!" (Do not take the money!), and only the associated key ("X" or "M") works. After a blank screen (200, 500, or 800 ms delay), they hear the tone indicating the outcome. After a short blank screen, they report the perceived time interval ("Schatting tijd") between their button press and the tone.

Participants were only permitted to press the keys after the cues disappeared. Once a key was pressed, a tone sounded, indicating that their action had impacted the balance. There was a delay between their keypress and the audio, varying between 200, 500, and 800 ms. After this, there was a blank screen for 500 ms, followed by the appearance of a slider ranging from 0 to 1000 ms (Fig. 3). This slider was employed instead of the manual note-taking approach used in previous research^4^. Participants were tasked with estimating the elapsed time between their keypress and hearing the tone. They were informed that the delays would fluctuate randomly between 0 and 1000 ms across trials. To avoid any initial response bias in their time estimations, the tooltip of the slider was hidden, so participants could freely navigate the slider to provide estimations. Once they provided their estimates, a black intertrial screen was displayed for 500 ms before transitioning to the next trial. The delays associated with the key presses were randomized evenly within each block. Before the primary task, participants underwent six practice trials. These were followed by brief task instructions and an attention check. The entire TIE task comprised 120 trials, split into two blocks of 60 trials. Every 20 trials were punctuated with a 30-s break.

### Data preparation and analysis

First, the practice trials were removed from the data, leaving 5400 experimental trials, 11 of which were marked as timeouts since the participants' button presses were more than 10 s after cue removal. These trials were excluded from the data, and the remaining trials were used to detect the participants who did not show a linear contrast with changing delays. To detect these participants, a linear trend analysis was performed for each participant with contrast coefficients of − 1, 0, and 1 for the three delays (200, 500, and 800 ms). In the end, 42 participants (27 female, M_age_ = 23.38) were included in the analysis (see Caspar et al.^4^, for a similar inclusion criterion).

As descriptive statistics, we calculated the frequency of participants' decisions to take or not take money from the other participant in free choice trials. Later, these frequencies were examined across both block orders (start free vs. start forced). To further assess whether the decision to take or not take money was independent of the block order, a chi-square test of independence was performed.

To investigate the effect of freedom of choice, outcome, and block order on the time estimations, a linear mixed-effects model (LMM) was fitted to the data, including freedom of choice, outcome, block order, and their interactions as fixed effects in the model. The LMM is especially appropriate to control for the unequal responses in free choice harm vs. no harm trial conditions (see below, the descriptive statistics about behavior), and averaging trials within conditions leads to unbalanced means and even empty cells. For the random effects, only the participant number was considered a random factor. All previously described analyses were conducted using R version 4.2.3^36^ and the lme4 package^37^.

Furthermore, we employed a mixed-model ANOVA using SPSS to explore perceived differences in autonomy as well as emotional experiences of enjoyment, struggle, and unpleasantness between the free and forced choice conditions. In addition to the within-subject factor of freedom of choice, the between-subject factor of block order was also included in the analysis. The results of the ANOVAs of emotional experiences can be seen in the Appendix.

### Supplementary Information

Supplementary Information.

## Data Availability

The data used in this study is publicly available and can be accessed via the following link; https://osf.io/gqmbe/?view_only. For any inquiries or additional information regarding the dataset, please email the corresponding authors.
