# Supplementary material for: Obedience induces agentic shifts by increasing the perceived time between own action and results
Source: Sci Rep. 2024 Jul 22;14:16769. doi: 10.1038/s41598-024-66499-8 (PMC11263708; doi:10.1038/s41598-024-66499-8)
Supplement: Supplementary file 1 — Supplementary Information. [file 41598_2024_66499_MOESM1_ESM.docx]

**Perceived Autonomy and Emotions**

**Enjoyment**

Using a mixed-model ANOVA in SPSS, we analyzed perceived enjoyment differences between the free and forced choice conditions, considering block order as a between-subject factor. There was no significant main effect of choice F (1, 40) = 2.57, *p* =.117, *η_p_^2^* = 0.06. The interaction between enjoyment measure and block order was also not significant, F(1, 40) = 0.68, *p* = 0.415, *η_p_^2^* = 0.02. The effect of block order on enjoyment was not significant, F(1, 40) = 0.57, *p* = 0.455, *η_p_^2^* = 0.01.

**Struggle**

A mixed model ANOVA with the experienced struggle scores revealed that the main effect of choice was not significant, F(1, 40) = 0.22, *p* =.640, η_p_^2^ = 0.01. Furthermore, the interaction between choice and block order also failed to reach significance, F(1, 40) = 1.47, *p* =.233, *η_p_^2^* = 0.04. The effect of block order was not significant either, F(1, 40) = 0.34, *p* =.565, *η_p_^2^* = 0.008.

**Unpleasantness**

A mixed model ANOVA with the experienced unpleasantness scores revealed that the main effect of choice was not significant, F(1, 40) = 1.58, *p* =.216, η_p_^2^ = 0.04. The interaction between choice and block order also failed to reach significance, F(1, 40) = 1.02, *p* =.319, η_p_2 = 0.03. The effect of block order was also not significant, F(1, 40) = 1.49, *p* =.229, η_p_^2^ = 0.04.

**Supplementary Analysis on TIE for First Block Responses**

For the sake of serving as a proxy for a between-subjects experimental design, we conducted a supplemental analysis focusing on the TIE for the first block responses (free vs. forced start). The results of this analysis indicated that there was no difference between free (M = 338.94, SD = 157.00) and forced choice (M = 369.40, SD = 113.54), t(40) = -0.72, *p* = .32, Cohen’s d = 0.22.offering an estimation of effect size in a between-subject design.
